# Prognostic Value of Blood Flow Measurements Using Arterial Spin Labeling in Gliomas

**DOI:** 10.1371/journal.pone.0099616

**Published:** 2014-06-09

**Authors:** Julia Furtner, Benjamin Bender, Christian Braun, Jens Schittenhelm, Marco Skardelly, Ulrike Ernemann, Sotirios Bisdas

**Affiliations:** 1 Department of Biomedical Imaging und Image-guided Therapy, Medical University of Vienna, Vienna, Austria; 2 Department of Neuroradiology, Eberhard Karls University, Tübingen, Germany; 3 Department of Neurology, Eberhard Karls University, Tübingen, Germany; 4 Department of Neuropathology, Eberhard Karls University, Tübingen, Germany; 5 Department of Neurosurgery, Eberhard Karls University, Tübingen, Germany; The Ohio State University Medical Center, United States of America

## Abstract

The period of event-free survival (EFS) within the same histopathological glioma grades may have high variability, mainly without a known cause. The purpose of this study was to reveal the prognostic value of quantified tumor blood flow (TBF) values obtained by arterial spin labeling (ASL) for EFS in patients with histopathologically proven astrocytomas independent of WHO (World Health Organization) grade. Twenty-four patients with untreated gliomas underwent tumor perfusion quantification by means of pulsed ASL in 3T. The clinical history of the patients was retrospectively extracted from the local database. Six patients had to be excluded due to insufficent follow-up data for further evaluation or histopathologically verified oligodendroglioma tumor components. Receiver operating characteristic (ROC) curves were used to define an optimal cut-off value of maximum TBF (mTBF) values for subgrouping in low-perfused and high-perfused gliomas. Kaplan-Meier curves and Cox proportional hazard regression model were used to determine the prognostic value of mTBF for EFS. An optimal mTBF cut-off value of 182 ml/100 g/min (sensitivity  = 83%, specificity  = 100%) was determined. Patients with low-perfused gliomas had significantly longer EFS compared to patients with high-perfused gliomas (p = 0.0012) independent of the WHO glioma grade. Quantified mTBF values obtained by ASL offer a new and totally non-invasive marker to prognosticate the EFS, independently on histopathological tumor grading, in patients with gliomas.

## Introduction

In spite of perpetual advancements in glioma treatment the prognosis of glial brain tumors, especially high-grade gliomas, remains poor with a mean progression free survival of about 16.5 and 6.8 months in anaplastic astrocytomas and glioblastomas respectively [1], [2]. The unequivocal standard of glioma grading and therefore predicting patient survival is still histopathology. However, a hardly negligible circumstance for histopathological grading is the tumor heterogeneity, which may lead to sampling errors [3]. Moreover, inter- and intra-pathologist variability may also lead to an inaccurate tumor evaluation [4], [5], [6]. Such sources of diagnostic inaccuracies in histopathology may be the reason for the observed variability of progression-free survival duration in patients with similar histopathological tumor grades. Therefore, additional diagnostic markers with implications for tumor recurrence and progression would be valuable. Under this scope and apart from molecular markers [2], different non-invasive, imaging markers have turned out to be predictive as well as prognosticative for progression-free survival in gliomas. Previously published data mainly focused on diffusion-weighted imaging reflecting tumor cell density as well as various perfusion techniques to reveal tumor vascularity, which are both amongst others essential features of malignancy [7]. The most popular and robust technique to depict tumor perfusion is still dynamic susceptibility-weighted contrast-enhanced MR imaging. Due to the fact, that this technique relies on the intravenous application of contrast media it could pose a challenge for people with allergic reactions to contrast media and for patients with impaired kidney function, in whom the application of gadolinium-based contrast media could, in rare cases, lead to nephrogenic systemic fibrosis [8].

Arterial spin labeling (ASL) is a totally non-invasive, perfusion-weighted MRI method that calculates cerebral perfusion maps without the need of intravenous contrast agent administration. Instead, water protons of the arterial blood in the feeding vessels of the brain are magnetically labeled and used as an endogenous tracer. The signal is produced after the subtraction of labeled imaging section from a control image without prior labeling. Moreover, ASL offers the possibility to quantify cerebral blood flow values, determining absolute perfusion values of each voxel in ml/g/min, by using a general kinetic model [9]. ASL perfusion is a reliable alternative to the routinely used dynamic susceptibility contrast perfusion technique [10], [11], [12], [13], [14] and benefits from a high test-retest reliability [15]. One of the most important drawbacks of ASL is that this perfusion technique is characterized by a moderate spatial resolution; however, the increased clinical availability of high-field MR scanner has helped to increase the signal-to-noise ratio [16]. Previously published papers indicate that ASL can make a significant contribution to preoperative glioma grading, however the merit of ASL for prognosticating event-free survival (EFS) has not been addressed. Our hypothesis was that maximum tumor blood flow (mTBF) values, quantified by means of preoperative ASL imaging might hold prognosticative value in patients with gliomas. Therefore, the purpose of this study was to correlate EFS with mTBF values in glioma patients and to examine any prognosticative value of the ASL technique independently to WHO tumor classification.

## Materials and Methods

### Ethics Statement

The Institutional Ethics Review Board of the University of Tübingen approved this prospective pilot study and written informed consent was obtained from all participants.

### Patients

From November 2010 to September 2011, 24 consecutive patients with new-onset glial tumors were recruited and underwent baseline ASL imaging. Of these, 6 patients had to be excluded because of missing follow-up data (n = 3) or histopathologically verified oligodendroglial tumor components (n = 3), which are known to possess a different perfusion pattern [17], [18]. The remaining 18 patients met the inclusion criteria of histopathologically confirmed primary astrocytomas without any prior brain tumor treatment, including corticosteroids, and any evidence of immune status compromise or other systemic malignancy. The study population was composed of 11 male and 7 female patients (mean age: 53.6 years; range: 31–78 years). The tissue specimens were obtained at stereotactic biopsy (n = 1) or surgical resection (n = 17). In general, a neuronavigation system was used intraoperatively to guide stereotactic biopsies toward the contrast-enhancing tumor parts, if evident in baseline MRI, based on the assumption that they reflect the most malignant tumor parts. The histological assessment, which was blind to initial diagnosis re-performed for the study purpose in order to detect any inter-reader bias, revealed 2 low-grade gliomas (diffuse astrocytomas - WHO grade II) and 16 high-grade gliomas (2 anaplastic astrocytomas - WHO grade III and 14 glioblastomas - WHO grade IV) and was based on the criteria of the “The 2007 World Health Organization Classification of Tumors of the Central Nervous System” [7].

### Treatment and Follow-up

Both low-grade gliomas were resected in total, as verified in subsequent follow-up MRIs, and had no further therapy. All high-grade gliomas received tumor resection to the greatest possible extent (total resection, n = 9; partial resection, n = 7) and adjuvant chemotherapy with temozolomide with concomitant radiation therapy (60 Gy) according to the Stupp schema. Total tumor resection was defined as resection without visual residual enhancing tumor portions based on intraoperative MRI images as well as MR images obtained <48 hours after surgery. On the contrary, partial resection was defined as incomplete tumor removal with residual contrast enhancing tumor tissue (detectable contrast enhancement on T1-weighted imaging with a volume of more than 0.175 cm^3^) on MRI images obtained <48 hours after surgery for example due to eloquent brain areas adjacent to tumor location [19], [20].

All patients were evaluated every 3 months (the follow-up period for patients with low-grade gliomas was extended to every 6 month in the second year). The follow-up evaluation was performed by a neuro-oncologist and included physical, neurologic and MRI examination. Tumor progression was defined as a new tumorous lesion and/or as an >25% increase in tumor size of contrast enhancing and/or non-contrast enhancing parts according to RANO criteria [21]. Transient increased edema and contrast enhancement that resolved or stabilized at the subsequent MRI examinations was assessed as pseudoprogression.

### Data acquisition

MRI examinations were performed on a 3T MRI scanner (Trio Tim; Siemens Medical Solutions, Erlangen, Germany), using a 32-channel head coil. The ASL sequence was added to the standard tumor MRI protocol, which consists of T2-weighted turbo-inversion recovery-magnitude (TIRM), fast spin-echo T2-weighted, diffusion-weighted images and 3D T1-weighted gradient-echo sequences before and after i.v. contrast medium application (0.1 ml/kg body weight of a gadolinium-based contrast agent).

ASL perfusion imaging was performed using a pulsed ultrafast echo planar imaging sequence with a single subtraction with thin-section TI1 periodic saturation (Q2TIPS) and a proximal inversion with control for off-resonance effects (PICORE) tagging scheme. We refer our readers to previous work for a detailed technique description [22]. The ASL imaging parameters were as follows: TR = 3000 ms, TE = 19 ms, field of view  = 240×240 mm, voxel size  = 3.75×3.75×5 mm, flip angle  = 90°, number of slices  = 9, slice thickness  = 5 mm, intersection gap  = 1 mm, T1 = 700 ms, saturation stop  = 1600 ms, T2 = 1800 ms, measurement repetitions  = 60. The acquisition time of the ASL sequence was 3 min and 40 s. Perfusion quantification was facilitated by acquisition of a single-shot echo-planar MR sequence without prior inversion. Crusher gradients were used to suppress the intravascular signal, which could lead to artificially high measurements when trying to quantify perfusion. The administration of contrast agent was unexceptionally performed after the acquisition of ASL data, due to the known T1 shortening effect of gadolinium-based contrast agents, which results in a reduction of the signal-to-noise ratio [23].

### Data analysis

ASL data were transferred for off-line post-processing. After movement correction and spatial smoothing (Gaussian kernel, width  = 5 mm) with SPM8 (Statistical Parametric Mapping, Wellcome Trust Centre for Neuroimaging), cerebral blood flow (CBF) maps were generated using in-house software in Matlab (MathWorks, Natick, MA). For the identification of the healthy white matter (WM), the 3D T1-weighted datasets were segmented into WM, gray matter (GM), and cerebrospinal fluid (CSF) by selection of all voxels that belonged to the WM (probability of >80%). Signal intensities due to edema or tumor tissue presented with <80% probability. CBF (in ml/100 g/min) was calculated by following the approach of Warmuth et al. [11]. 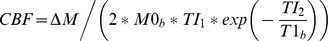



ΔM is the magnetization difference between control and tag images; TI_1_ is the width of the tag bolus; TI_2_ = TI_1_+w the inversion time of the actual slice with a post-labeling delay till slice acquisition of w; T1_b_ is the constant relaxation time of arterial blood, which was estimated to be 1490 ms. M0_b_ the signal of a voxel containing 100% fully relaxed blood. It was estimated as previously described [11], [24] by
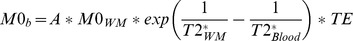



For the proton density A = 1.06, M0_WM_ is the signal intensity in the M0 image, T2*_WM_ = 40 ms and T2*_Blood_ = 80 ms at 3T. Co-registered T1 post-contrast images were used for detection of tumor location and spatial extent of non-enhancing and contrast-enhancing tumor parts. Regions of interest (ROIs) in tumor were manually drawn on each slice of the ASL sequence by two experienced neuroradiologists in consensus, blinded to tumor histopathology. Areas with extended necrosis were spared. The mean tumor volume, measured as contrast-enhancing tissue in gliomas with disrupted blood-brain-barrier or as signal abnormality in T2-weighted imaging in unenhancing gliomas was 32.6 cm^3^ (range: 1.4–76.7 cm^3^). The maximum TBF (mTBF) value in each ROI was recorded.

### Statistical analysis

The endpoint of interest was considered to be event-free survival (EFS) defined as the time (in days) between tumor surgery and progression/death or the last follow-up visit in case of absent tumor progression/death. Receiver operating characteristic (ROC) analysis was used to assess the optimal (showing the highest specificity/sensitivity) cut-off value of mTBF for prognosticating tumor progression. This value was determined to group all gliomas in high-perfused or low-perfused ones regardless of their histopathology. Kaplan-Meier survival curves according to this criterion were plotted. The result of the logrank test for the comparison between the survival curves was used to decide whether the grouping variable has a significant influence on survival time. The independent samples t-test to compare the average mTBF of the two independent samples with correction for unequal variances (Welch test) was applied. Finally, multivariate Cox proportional-hazards regression was used to analyze the significance of mTBF, histological grade and resection (total vs. partial) as risk factors on EFS. For all statistical tests, performed by MedCalc (12.7.5 for Windows, MedCalc Software, Mariakerke, Belgium), *P*-values <.05 were considered significant and their interpretation was explorative.

## Results

**Figure 1:**
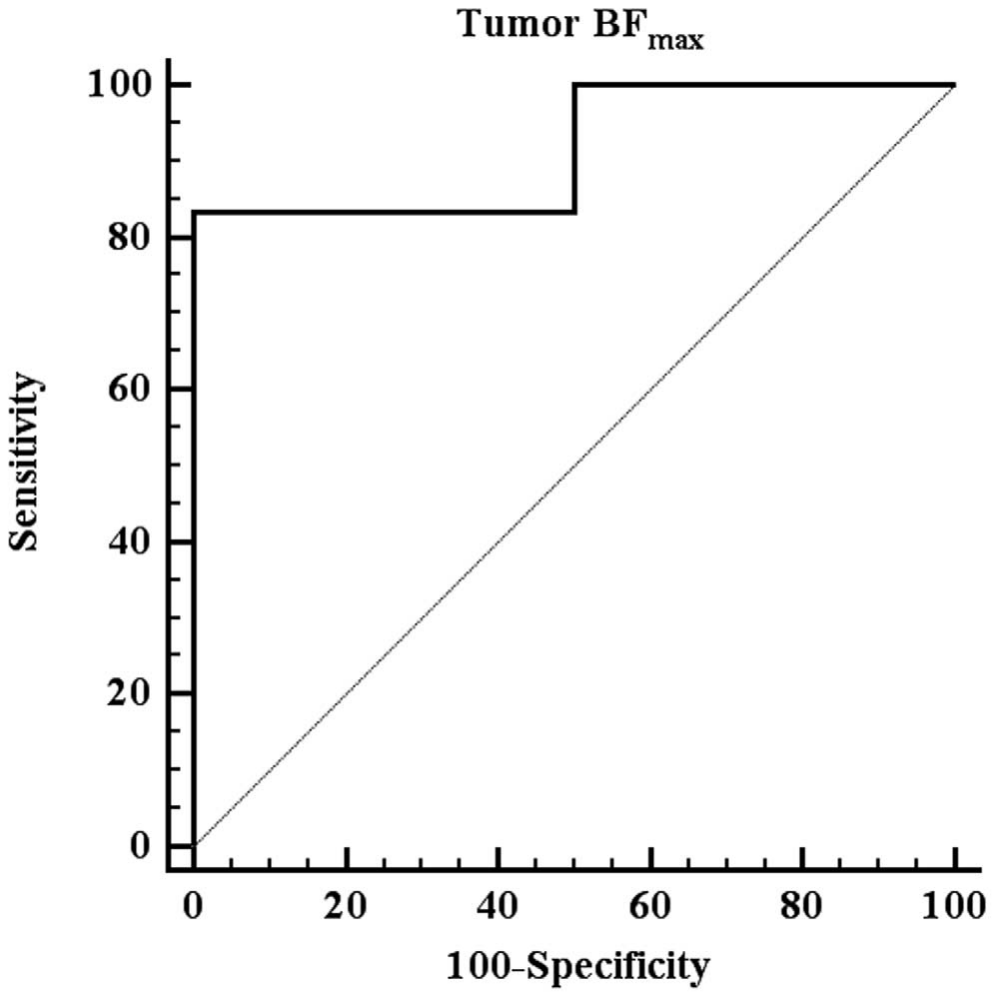
ROC curve analysis of mTBF for prognosticating disease progression (optimal cut-off value: mTBF = 182 ml/100 g/min, sensitivity  = 83%, specificity  = 100%, AUC = 0.94).

**Figure 2:**
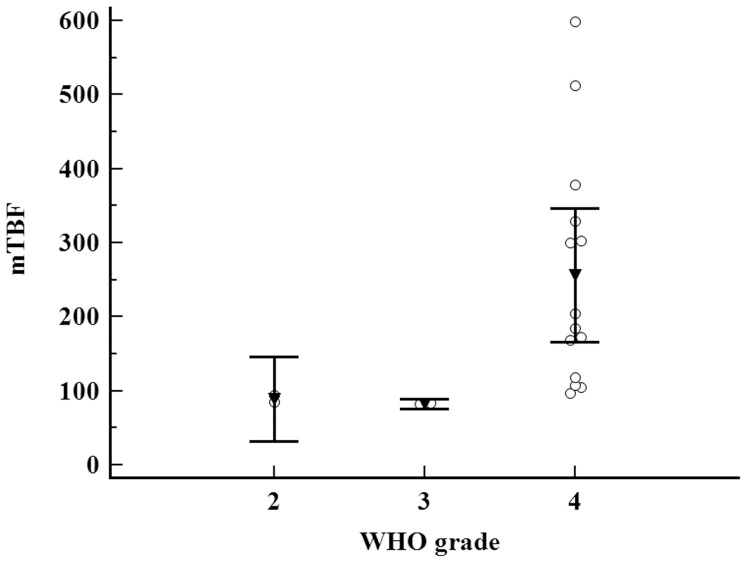
Multiple comparison graph illustrating the average mTBF of the examined tumors according to their WHO grade. All data are plotted by dots (circles), the mean values in each group by triangles, and the 95% CI by bars. mTBF is measured in ml/100 g/min.

The mTBF values of all gliomas ranged from 82–599 ml/100 g/min (mean: 218±155.02 ml/100 g/min). The optimal mTBF cut-off value for prognosticating tumor progression was 182 ml/100 g/min (sensitivity 83%, specificity 100%, area under the curve (AUC) 0.94) (**Fig. 1**). Tumors with mTBF≤182 ml/100 g/min were referred as low-perfused gliomas (n = 8), whereas tumors with mTBF>182 ml/100 g/min were classified as high-perfused gliomas (n = 10). The histopathological grades among the group of low-perfused tumors included diffuse astrocytomas (WHO grade II) (n = 2), anaplastic astrocytomas (WHO grade III) (n = 2) as well as glioblastomas (WHO grade IV) (n = 4), whereas the group of high-perfused tumors consisted entirely of glioblastomas. The distribution of the average mTBF according to WHO grade is illustrated in **Fig. 2**.

**Figure 3:**
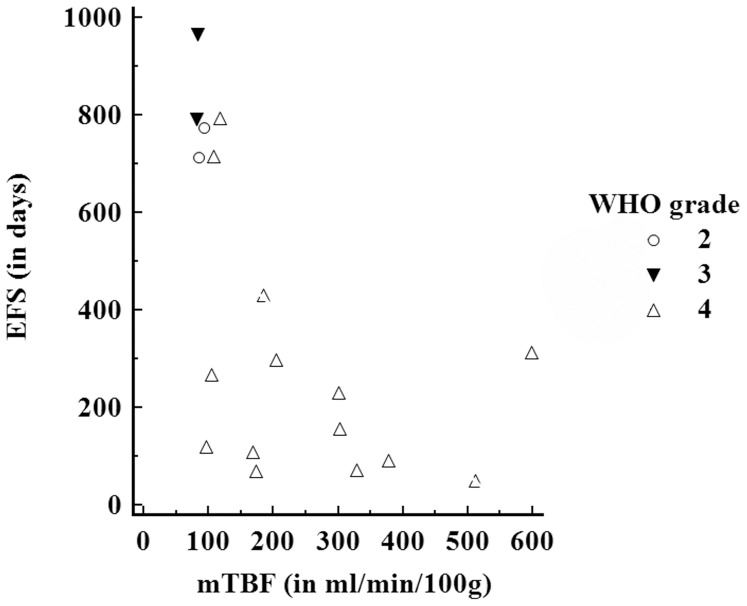
Scatterplot demonstrating the EFS over mTBF for all patients including the WHO grade of each case.

**Figure 4:**
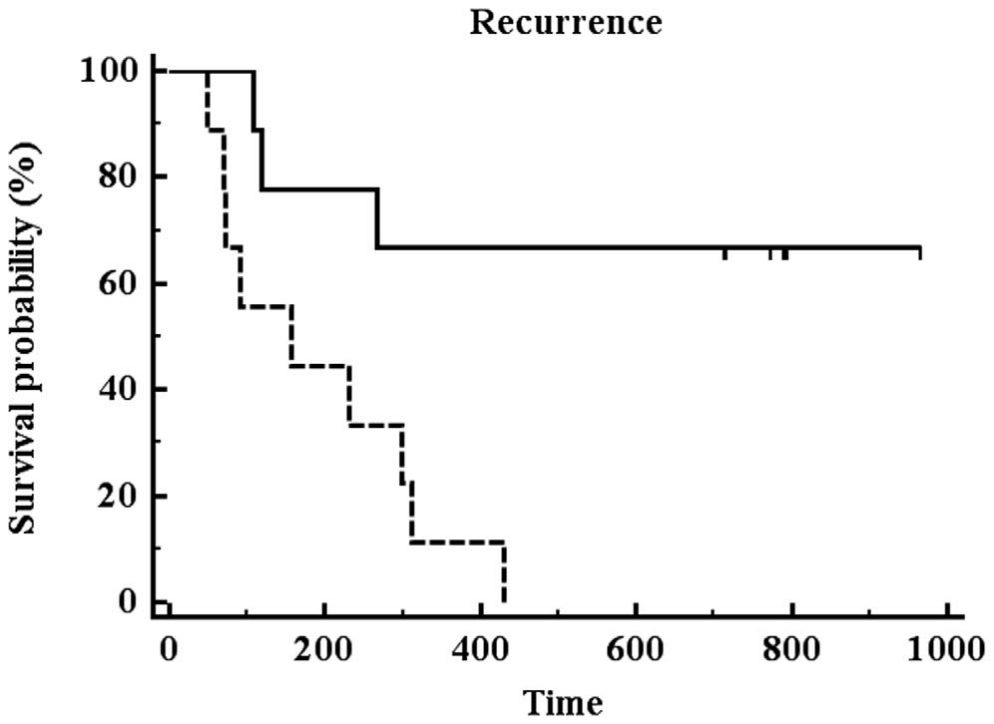
Kaplan-Meier survival curve analysis for patients with low-perfused (continuous line) or high-perfused (dashed line) gliomas (pooled patients irrespectively of tumor surgery). The patients were stratified in two groups according to the optimal mTBF cut-off value from the ROC curve analysis. Patients with low-perfused gliomas had significantly longer EFS compared to patients with high-perfused gliomas (*P* = 0.0012).

EFS and mTBF is demonstrated on an individual case basis in **Fig 3**. EFS and average mTBF values for low-perfused and high-perfused gliomas are presented in **Table 1**. The mean follow-up period was 386 days (range 49–1010 days). All high-perfused gliomas suffered either tumor progression or death (progression: n = 9; death: n = 1) during the follow-up. In contrast, only 2 (25%) out of 8 patients with low-perfused gliomas showed tumor progression and none of them died. The mean EFS period in patients with low-perfused gliomas accounted for 772.5±290.9 days (95% CI: 539.3–1006 days) compared to 181.8±129.8 days (95% CI: 105.5–258.1 days) in high-perfused gliomas (logrank test: *P* = 0.0012) (**Fig. 4**). Notably, 2 glioblastoma patients (IDH, MGMT positive and IDH negative, MGMT positive, respectively) without residual tumor after resection presented with low blood flow (mTBF<100 ml/100 g/min). These patients had EFS periods of 716 and 794 days, respectively, which were remarkably longer than the mean EFS of WHO grade 4 cases in our cohort (183.7±121.7 days, 95% CI: 106.4–261 days).

**Table 1:** Descriptive statistics and comparison of EFS and mTBF values for low-perfused and high-perfused gliomas.

	N	EFS	mTBF
Low-perfused gliomas	8	772.5±290.9*	104.4±26.8**
High-perfused gliomas	10	181.8±129.8*	331.5±146.1**

*P = 0.0012;

**P = 0.0013.

N: number of patients; EFS: mean of event-free survival in days ± SD; mTBF: average of mTFB values in ml/100 g/min ± SD.

**Figure 5:**
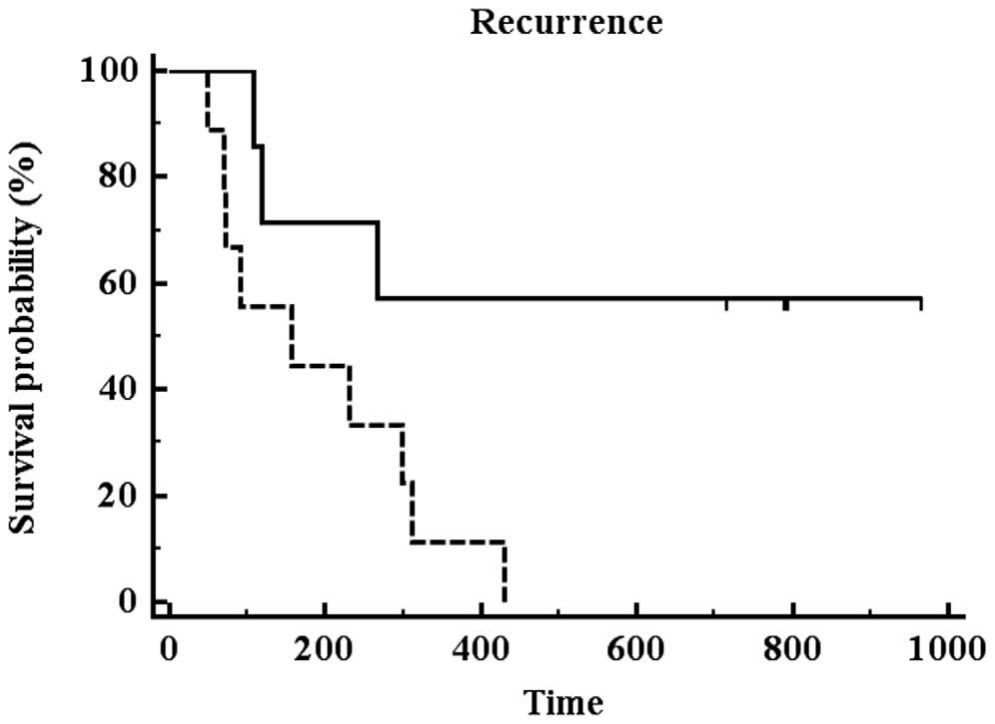
Kaplan-Meier survival curve analysis for patients with low-perfused (continuous line) or high-perfused (dashed line) gliomas (stratified according to the optimal mTBF cut-off value) after total tumor resection. Patients with low-perfused gliomas had significantly longer EFS compared to patients with high-perfused gliomas (*P* = 0.0046).

**Figure 6:**
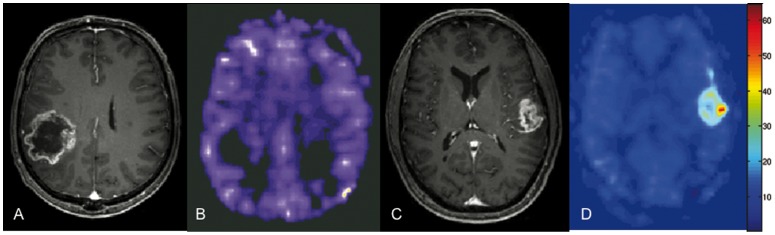
Contrast enhanced T1-weighted images (a, c) with corresponding ASL perfusion maps (b, d) in two glioblastoma patients. Figs. 4a and 4b show a low-perfused (mTBF = 97 ml/100 g/min) tumor, Figs. 4c and 4d depict a high-perfused (mTBF = 599 ml/100 g/min) glioma.

The multivariate Cox proportional-hazards regression (overall model fit: *P* = 0.0005) revealed that only resection (partial, n = 7 vs. total, n = 11) was significant prognosticator (*P* = 0.04) for tumor recurrence, while mTBF demonstrated a tendency to significance (*P* = 0.092). In order to exclude any bias due to the extent of surgery, we re-conducted the Kaplan-Meier curve analysis including only patients with total tumor resection (n = 12); among them 7 low-perfused and 5 high-perfused gliomas. Despite the lower sample size, the comparison revealed again significantly (*P* = 0.0046) longer EFS for patients with low-perfused gliomas (average mTBF: 866.1±92.5 days (95% CI: 684.9–1001 days)) compared to patients with high-perfused gliomas (average mTBF: 261.3±65.4 days (95% CI: 133.0–389.1 days)) (**Fig.5**). Examples of ASL perfusion maps and corresponding T1-weighted post-contrast images for low- and high-perfused glioblastomas are presented in **Fig. 6**.

## Discussion

In contrast to studies using dynamic susceptibility-weighted contrast-enhanced perfusion-weighted images to obtained relative perfusion data, the substantial contribution of this work design is the usage of ASL to obtain absolute MR perfusion values. The absence of intravenous application of any contrast agent makes ASL appealing for patients with elevated serum creatinin and previous allergic reaction to gadolinium. However, ASL suffers from low spatial resolution and limited brain tissue coverage, making them the most relevant shortcomings of the method. One the other hand, ASL benefits from the increasingly available high-field MR scanners resulting in an increased perfusion signal [16].

The results of our study indicate that baseline mTBF, quantified by ASL, is a significant prognostic factor in patients with gliomas. These results are in essential agreement with previously published data in perfusion-weighted MRI. Bisdas et al. suggested that maximal relative cerebral blood flow is a stronger predictor of 1-year survival compared to histopathology in patients with astrocytomas [18]. Accordingly, Law et al. revealed the predictive value of relative CBF for the progression-free survival in low-grade and high-grade gliomas, independently on histopathology [25], [26]. Also, studies exclusively focusing on high-grade glioma postulated the utility of perfusion-weighted images in predicting tumor progression and patient survival [1], [27]. Although multivariate Cox proportional-hazard regression revealed that tumor histopathology and mTBF did not act as independent prognostic factors in our study, there was, similar to previously published data, a tendency for mTBF to be superior in the prognosis of EFS compared to tumor histopathology. The missing significance could be the result of the low number of study participants.

Warmuth et al. used ASL data quantification for the differentiation between high-grade and low-grade gliomas [11]. The provided mTBF values (low-grade gliomas 65–337 ml/100 g/min; high-grade gliomas 140–885 ml/100 g/min) are in accordance to our results (low-grade gliomas 85–94 ml/100 g/min; high-grade gliomas 82–599 ml/100 g/min). Noteworthy, both studies show a wide range of perfusion values, mainly within the high-grade gliomas subgroup, and an overlap in perfusion values between high-grade and low-grade gliomas. Thus, we subdivided our patient population in low-perfused and high-perfused gliomas (mTBF cut-off value = 182 ml/100 g/min) regardless of the WHO tumor classification. We found that patients with low-perfused gliomas had a significantly longer time to tumor progression in contrast to patients with high-perfused gliomas, irrespectively of histopathologic tumor grading. To demonstrate that this circumstance persists also in case of excluding other well-known prognostic factors we checked our dataset for uneven dispersion of other well known prognosticative values except of histopathological features. An inhomogeneous distribution of gross tumor surgery in low-perfused gliomas (87.5%) and high-perfused gliomas (40%) could be revealed. In order to exclude any bias in the Kaplan-Meier curves from the extent of surgery, we re-conducted the analysis including only patients with total tumor resection. Despite the lower sample size, significantly longer EFS for patients with low-perfused gliomas compared to patients with high-perfused gliomas was observed.

Therefore, consideration should be given to a prognostic grading system based on imaging data, especially focusing on tumor perfusion, in addition to histological tumor grading. An outcome-based astrocytoma grading system would have the advantage of heading towards a more individual therapeutic scheme. Law et al. postulated that in histologically classified low-grade gliomas the addition of perfusion values could have consequences at the time of tumor surgery [25]. In high-grade gliomas, where an immediate surgery followed by adjuvant radio-chemotherapy is the customary procedure, an additional anti-angiogenic therapy could possibly be beneficial for patients with high-perfused tumor components. Moreover, recently published data indicated that perfusion values may prognosticate tumor response to radiation therapy and therefore, they may be used to for dose-painting of heterogeneous tumors, which might benefit from dose escalation [28].

The small sample size, especially regarding low-grade gliomas, is an important limitation of the present work. This is particularly due to lower overall incidence of low-grade gliomas in contrast to high-grade gliomas [29], and due to strict patient selection criteria, on account of missing follow-up data or histopathologically verified oligodendroglial tumor components, which are known to show longer survival rates despite high perfusion values [1], [17], [18]. The exploratory nature of our study suggests that high-grade tumors may present with low perfusion and thus ASL-based blood flow may possess prognostic value, which has to be compared against established clinical (Karnofsky Performance Status) and molecular (MGMT, IDH) markers in larger patient populations.

## Conclusions

In conclusion, ASL offers a totally non-invasive, easily in routine protocol implemented MR perfusion technique. Moreover, mTBF derived from pre-treatment ASL images may be a useful imaging biomarker for predicting time to tumor progression, independently on histopathological tumor grading in glioma patients. As add-on to histopathology, MR-perfusion values may improve individual therapy planning.
